# Predicting prognosis and immunotherapy response among colorectal cancer patients based on a tumor immune microenvironment-related lncRNA signature

**DOI:** 10.3389/fgene.2022.993714

**Published:** 2022-09-07

**Authors:** Chuling Hu, Du Cai, Min-Er Zhong, Dejun Fan, Cheng-Hang Li, Min-Yi Lv, Ze-Ping Huang, Wei Wang, Xiao-Jian Wu, Feng Gao

**Affiliations:** ^1^ Department of Colorectal Surgery, The Sixth Affiliated Hospital, Sun Yat-sen University, Guangzhou, China; ^2^ Guangdong Institute of Gastroenterology, Guangzhou, China; ^3^ Guangdong Provincial Key Laboratory of Colorectal and Pelvic Floor Diseases, The Sixth Affiliated Hospital, Sun Yat-sen University, Guangzhou, China; ^4^ Department of Gastrointestinal Endoscopy, The Sixth Affiliated Hospital, Sun Yat-sen University, Guangzhou, China; ^5^ Department of Pathology, The First Affiliated Hospital of Anhui Medical University, Hefei, China

**Keywords:** colorectal cancer, tumor microenvironment, tumor immune microenvironment, long non-coding RNA, immunotherapy

## Abstract

Long non-coding RNAs (lncRNAs) remodel the tumor immune microenvironment (TIME) by regulating the functions of tumor-infiltrating immune cells. It remains uncertain the way that TIME-related lncRNAs (TRLs) influence the prognosis and immunotherapy response of colorectal cancer (CRC). Aiming at providing survival and immunotherapy response predictions, a CRC TIME-related lncRNA signature (TRLs signature) was developed and the related potential regulatory mechanisms were explored with a comprehensive analysis on gene expression profiles from 97 immune cell lines, 61 CRC cell lines and 1807 CRC patients. Stratifying CRC patients with the TRLs signature, prolonged survival was observed in the low-risk group, while the patients in the high-risk group had significantly higher pro-tumor immune cells infiltration and higher immunotherapy response rate. Through the complex TRLs-mRNA regulation network, immunoregulation pathways and immunotherapy response pathways were found to be differently activated between the groups. In conclusion, the CRC TRLs signature is capable of making prognosis and immunotherapy response predictions, which may find application in stratifying patients for immunotherapy in the bedside.

## Introduction

Colorectal cancer (CRC) ranks as the third most common cancers and the second most common cause of cancer-related deaths worldwide (Siegel et al., 2019), and the overall 5-years relative survival rate for CRC patients is approximately 64% (Miller et al., 2019). Though significant advances have been made in the treatment of CRC, the recurrence rate remains high in patients received standard chemotherapy and surgery (Ahiko et al., 2021; Fang et al., 2021; Kim et al., 2021). Recently, immunotherapy has emerged as a novel treatment approach and achieved exciting results in some cancer types (Doki et al., 2022; Makker et al., 2022; Schmid et al., 2022). For CRC, anti-programmed death 1 (anti-PD-1) antibodies, such as pembrolizumab and nivolumab, and CTLA-4 inhibitor ipilimumab were approved by FDA (Pan et al., 2021). Though a subset of patients with mismatch repair deficiency or high microsatellite instability CRC benefit a lot from immune checkpoint blockade therapy (Pan et al., 2021), the overall response rate of immunotherapy remains low in all cases of CRC and there were difficulties in stratifying suitable patients for immunotherapy (Chen et al., 2021). Widely used biomarkers for immunotherapy response prediction, such as impaired DNA mismatch repair deficiency and microsatellite instability (MSI) (Cortes-Ciriano et al., 2017), only have moderate accuracy, and there are still a portion of CRC patients with MSI/mismatch repair deficiency tumors do not respond to the treatment (Gibney et al., 2016; Cohen et al., 2020). Therefore, it is of vital importance to develop effective methods to predict CRC prognosis and immunotherapy response.

In recent years, tumor microenvironment (TME) was identified to have an huge impact on the behavior and characteristics of cancer (Li et al., 2007). TME is made up of noncellular components, such as extracellular matrix and types of signaling molecules, and non-tumor cellular components, including epithelial, smooth muscle, immune cells and other types of cells in the tumor niche (Li et al., 2007; Valkenburg et al., 2018). The crosstalk between tumor cells and non-tumor cells was found taking an active part in regulating the development and therapeutic responses of cancer (Zhang et al., 2020a). Among cells of TME, different types of tumor infiltrating immune cells build up tumor immune microenvironment (TIME). Tumor infiltrating lymphocytes, such as B cells, CD4 positive T helper cells, CD8 positive cytotoxic T lymphocytes and regulatory T cells (Tregs), are communicating and cooperating with other tumor infiltrating immune cells including macrophages, natural killer cells and dendritic cells (Zhang et al., 2020b). Significantly influencing the survival and the immunotherapy response of patients (Zhang et al., 2020b), TIME is essential in the progress and the treatment of CRC.

Defined to be non-coding RNAs longer than 200 nucleotides in length (Cao, 2014), long non-coding RNA (lncRNAs) are important regulators of multiple biological processes, including cell proliferation (Xiong et al., 2019), apoptosis (Huang et al., 2019), differentiation, tumorigenesis (Bhan and Mandal, 2014), metastasis (Tian et al., 2019), cell cycle regulation (Wu et al., 2018), epithelial-mesenchymal transition (Wang et al., 2019a) and drug resistance (Wei et al., 2019) by forming RNA-RNA, RNA-DNA, RNA-protein interactions and serving as competing endogenous RNAs (ceRNA) in a variety of regulatory mechanisms (Yao et al., 2019). Actually, emerging evidence has implicated that lncRNAs are key coordinators and regulators within tumor infiltrating immune cells that build up the complex “ecosystem” of TIME, associating with recruitment, infiltration, differentiation, activation and pro-/anti-tumor function in those infiltrating immune cells (Sage et al., 2018; Xu et al., 2019; Zhang et al., 2020b; Zhang et al., 2021). By mediating and regulating important mechanisms and processes of immune response in the microenvironment (Bhan and Mandal, 2014; Zhou et al., 2019), lncRNAs within the tumor infiltrating immune cells occupy a central role in immunity regulation of the TIME, as well as in the development, progression and maintenance of many human tumors (Denaro et al., 2019), suggesting that TIME related lncRNAs (TRLs) could be potential diagnostic markers and therapeutic targets in CRC.

In this study, we developed a prognostic TRLs signature for prognosis and immunotherapy response predictions. The performance of model was validated with multiple independent cohorts, proving its potential to serve as a reliable predictor for patient survival and an indicator for immunotherapy.

## Materials and methods

### Data collection

#### Datasets of colorectal cancer cases

Transcriptome and clinical data of CRC cases were obtained from the Gene Expression Omnibus (GEO database, https://www.ncbi.nlm.nih.gov/geo/). Data collected from GEO was analyzed by Affymetrix Human Genome U133 2.0 Plus GeneChip Set platform. Clinical information and transcriptional profiles were downloaded from The Cancer Genome Atlas (TCGA, https://portal.gdc.cancer.gov/). Finally, excluding cases with incomplete clinical information, 519 cases of GSE39582 (Marisa et al., 2013) served as training cohort, 595 cases of TCGA CRC and 693 cases of GSE14333 (Jorissen et al., 2009), GSE17538 (Smith et al., 2010; Freeman et al., 2012; Williams et al., 2015; Chen et al., 2019), GSE33113 (de Sousa et al., 2011; Kemper et al., 2012), GSE37892 (Laibe et al., 2012) and GSE39084 (Kirzin et al., 2014) were used as two independent testing cohorts. The summary of clinical information of the three cohorts was shown in Table 1.

**TABLE 1:** Clinical characteristics of training and testing cohorts.

	Training	Testing 1	Testing 2
Age			
<65 y	192	243	293
≥65 y	326	352	400
Sex			
Female	233	273	334
Male	286	322	359
Location			
Left	310	335	227
Right	209	260	188
TNM Stage			
I	32	103	77
II	253	216	348
III	200	174	239
IV	34	83	29
MMR			
MSI	71	181	52
MSS	405	411	137
CMS Subtype			
CMS1	86	65	138
CMS2	215	196	248
CMS3	62	64	109
CMS4	112	110	145

Trianing: cohortGSE39582.

Testing cohort 1: TCGA-COAD and TCGA-READ.

Testing cohort 2: GSE14333, GSE17538, GSE33113, GSE37892 and GSE39084.

Location: location of tumor [right colon or left colon (rectum included)].

MMR, mismatch repair; MSI, microsatellite instability; MSS, microsatellite stable; CMS, consensus molecular.

#### Datasets of immune cell lines and colorectal cancer cell lines

Representing 17 different immune cell types, transcriptional profiles of 97 non-treated immune cell lines of healthy volunteers are collected from GEO database (Supplementary Table S1). Transcriptional profiles of 61 CRC cell lines were obtained from Cancer Cell Line Encyclopedia (CCLE, https://sites.broadinstitute.org/ccle/datasets) project. The downloaded transcription profiles of immune cell lines and CRC cell lines were all originally analyzed by Affymetrix Human Genome U133 2.0 Plus GeneChip Set platform.

#### Data preprocessing

**FIGURE 1:**
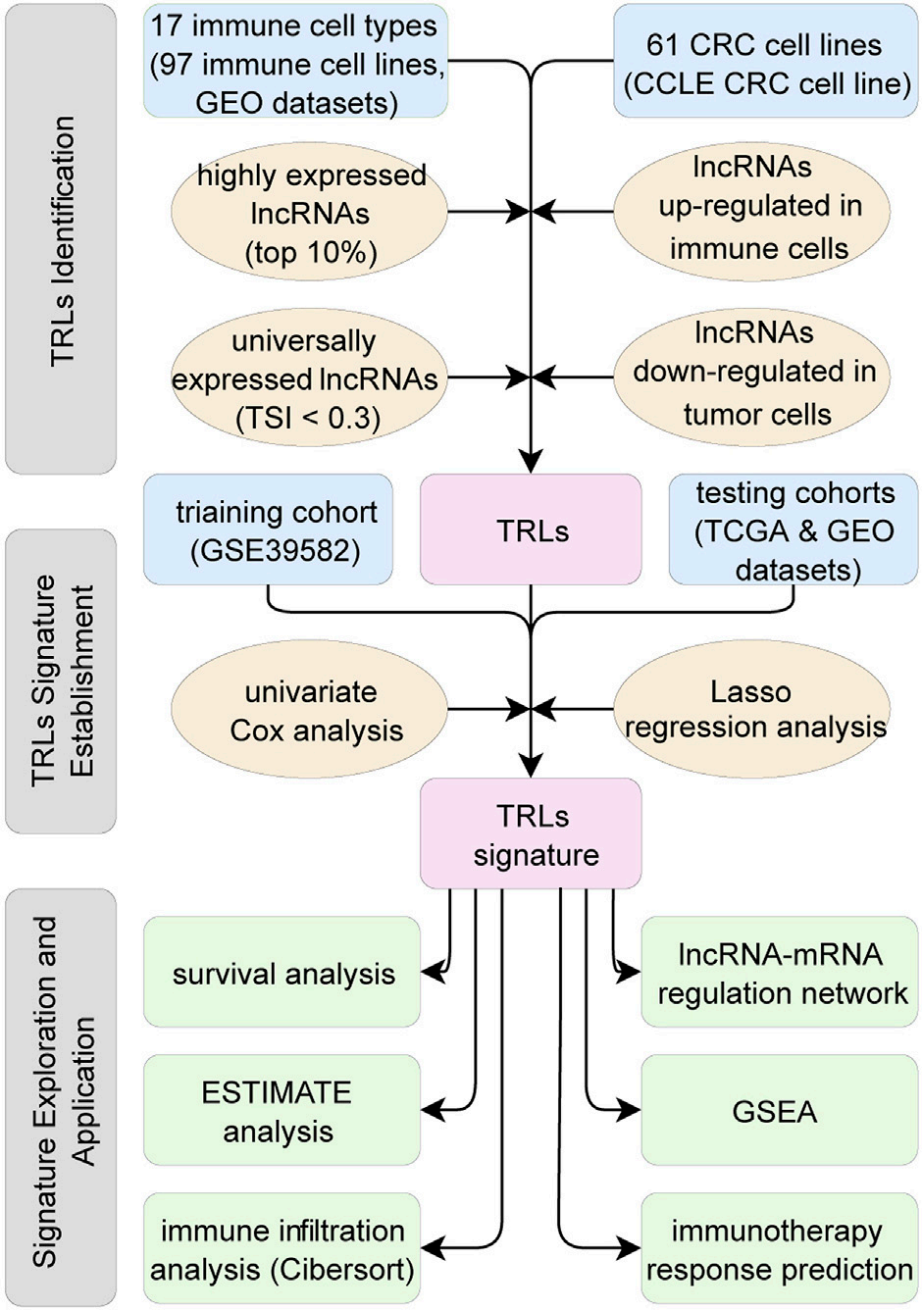
Flow chart of this study. First, TRLs of the CRC were identified. Second, TRLs signature was established utilizing Lasso regression. Third, TRLs signature was assessed on independent datasets and the related biological mechanisms were explored.

The downloaded GEO and CCLE transcriptional profiles were based on the Affymetrix Human Genome U133 2.0 Plus GeneChip Set. Probe information of the chip was reannotated by NetAffx Annotation Files (HG-U133_Plus_2 Annotations release 36, https://www.affymetrix.com/support/technical/byproduct.affx?product=hg-u133-plus), Gencode files (Long non-coding RNA gene annotation release 38, https://www.gencodegenes.org/human/) and Refseq files (Refseq H_sapiens annotation, https://ftp.ncbi.nlm.nih.gov/refseq/H_sapiens/annotation/) to find out probes that matched long non-coding RNAs, which were labeled as “lncRNA” in Gencode or “long non-coding RNA” in Refseq. Among 50,000 probes of the gene chip, only 2,287 probes had Ensembl ID or Refseq ID annotated as “lncRNA” or “long non-coding RNA”, which corresponded to 1892 unique lncRNA Ensembl IDs. Similar methods were also applied on the transcriptional profiles of TCGA to obtain the lncRNAs and their expression profiles. Finally, the shared 1724 lncRNAs were identified and the corresponding lncRNA expression matrixes were therefore established (Figure 1).

### Prognostic TRLs signature development

#### Identification of TRLs

The following three steps built up a workflow for identifying TRLs. First, utilizing the gene transcription profiles of immune cells, the top 10% expressed lncRNAs in each immune cell type were identified as immune-related lncRNAs. Second, tissue specificity index (TSI) (Yanai et al., 2005) was calculated across 17 immune cell types to identify universally expressed immune-related lncRNAs in all the immune cells of TIME. Ranging from 0 to 1, the smaller the TSI is, the more consistent the particular lncRNA expressing across all types of immune cells. Here, lncRNAs with TSI smaller than 0.3 were selected, so that universally high-expressed lncRNAs in all types of immune cells were identified. Third, using limma package (Ritchie et al., 2015), lncRNAs upregulated in immune cell lines and downregulated in CRC cell lines (logFoldChange >1.0 and adjusted *p* < 0.05) were recognized as lncRNAs expressing in immune cells rather than in the tumor cells. In other words, these lncRNAs were mainly expressed in the TIME, which were regarded as TIME related lncRNAs. Step by step, universally high-expressed lncRNAs in the immune cells of TIME were identified, namely TRLs in this study (Figure 1).

#### Development and validation of the TRLs signature

The prognostic value of each TRL was evaluated by univariate Cox proportional hazards regression analysis with the training set. TRLs with *p* < 0.1 were selected as candidates for the construction of the signature. Utilizing the LASSO regression analysis (Supplementary Figure S1), the TRLs signature was established based on the training cohort, and the risk score formula was generated as follows $Risk\;score=\underset{i}{\sum }Coefficient\left({TRL}_{i}\right)*Expression\left({TRL}_{i}\right)$ Considering their risk scores, patients were divided into low-risk group and high-risk group with a cutoff value calculated by Youden index. Using the survival package (Terry, 2022) and survminer package (Kassambara et al., 2021), the Kaplan-Meier survival curve combined with log-rank test was used to compare the survival difference between the two groups. Using the same cutoff value, the prognostic value of the lncRNA signature was further investigated in two independent testing cohorts (Figure 1).

#### Independent prognostic role of the TRLs signature

To investigate whether the signature could be independent of other clinical parameters, including risk group, age, sex, stage, location of the tumor, microsatellite stability (MSS) or microsatellite instability (MSI) status and consensus molecular subtypes (CMS) (Guinney et al., 2015), univariate and multivariate Cox regression analyses were performed, and *p* < 0.05 were considered as statistically significant.

### Differentially expressed gene (DEG) analys and gene set enrichment analysis (GSEA)

DEGs between the low-risk group and high-risk group were identified using the Limma package with age, sex and TNM stage factors adjusted (Ritchie et al., 2015). DEGs were visualized with pheatmap package in R. Log-fold-change > 0.5 and adjusted *p*-value < 0.05 were cutoff value for DEG analysis. Based on the results of DEG analysis and gene set collections of Molecular Signatures Database (MSigDB), GSEA was performed with clusterProfiler package (Yu et al., 2012) and HTSanalyzeR2 package (Wang et al., 2011). Pathways and gene sets from “curated gene sets” collection (C2), “ontology gene sets” collection (C5) and “immunologic signature gene sets” collection (C7) are used to perform the GSEA.

### The TRLs signature lncRNA-mRNA regulation network

LncRNAs associated RNA interactions, which included information about lncRNAs and their target mRNAs in the regulatory network, were collected from four different manually-curated and experimentally-supported RNA databases, including starBase v2.0 (Li et al., 2014), LncACTdb 2.0 (Wang et al., 2019b), LncTarD (Zhao et al., 2020) and LnCeCell (Wang et al., 2021). Over 1,000 pairs of lncRNA–target mRNA involving lncRNAs in the TRLs signature were selected. Spearman correlation analysis were applied to calculate the correlation coefficients between the expression of 10 lncRNAs of the signature and the expression of their target mRNAs based on transcription profiles of immune cell lines. Selecting the top 30 most correlated target mRNAs for each lncRNA, a regulatory network of the TRLs signature was constructed and visualized with Cytoscape software (version 3.8.2). The correlated target mRNAs were analyzed with GSEA to find out the targeted pathways, process of which was the same as above.

### Tumor immune infiltration analysis

Tumor purity and the infiltration level of stromal cells (StromalScore) and immune cells (ImmuneScore) were estimated by ESTIMATE package (Yoshihara et al., 2013). The fraction of tumor infiltrating immune cells in each sample, such as B cells, T cells, dendritic cells, macrophages, neutrophils and so on, were estimated by CIBERSORT algorithm (Newman et al., 2019). The fractions of 22 types of tumor infiltrating immune cells were calculated by Cibersort algorithm. Among them, nine types of immune cells playing important roles in the effect and regulation of the tumor immunology, including different types of T cells, NK cells and macrophages, were chosen to displayed in the figure. The correlations between risk score and StromalScore, ImmuneScore, tumor purity, fractions of immune cells were explored to identify whether the TRLs signature could be a reliable indicator in the CRC TIME.

### TRLs signature in immunotherapy response prediction

The expression level of immune checkpoint blockade therapy associated genes, such as PD-1 (PDCD1), PD-L1 (CD274), PD-L2 (PDCD1LG2), are closely related to the response of immunotherapy. The correlations between risk score and the expression of those key genes were investigated (*p* < 0.05). GSE165252, a dataset containing immunotherapy response information and transcriptional profiles of pre-treatment CRC tissues, was download from GEO and served as an external dataset to verify the TRLs signature’s capacity of predicting immunotherapy treatment response. Receiver operating characteristic (ROC) curve was therefore performed and the area under the ROC curve (AUC) was also calculated by pROC package.

### Statistical analysis

All statistical analyses were performed with R (version 4.1.0). T tests and Wilcoxon tests were performed for differential gene expression analyses and differential immune cell infiltration analysis. The Kaplan-Meier survival curve with log-rank test was used to compare the survival difference between the two groups. Univariate and multivariate Cox regression models were utilized to validate the prognosis value of the TRLs signature and other clinical parameters in patients of CRC. Pearson correlation analysis were applied to perform the correlation analyses of the study. DeLong test was employed to calculate the confidence intervals for the AUC values of the ROC curves.

## Results

### The construction of prognostic TRLs signature

Clinical data and gene expression data of 1807 CRC patients from multiple datasets were collected and divided into three cohorts (Table 1). A total of 1724 unique lncRNAs were identified from downloaded transcription profiles, and 60 lncRNAs were found to be universally high-expressed in the immune cells of TIME. Among the 60 TRLs, 18 lncRNAs were found to be prognostic markers for the survival of CRC patients and were selected for the construction of the signature. Using LASSO regression analysis (Supplementary Figure S1), a 10 TRLs signature was established, and the risk score of each patient was calculated. The corresponding coefficients of the TRLs were listed in the Supplementary Table S2. The flowchart of the whole study was showed in Figure 1.

### The prognostic value of the TRLs signature

**FIGURE 2:**
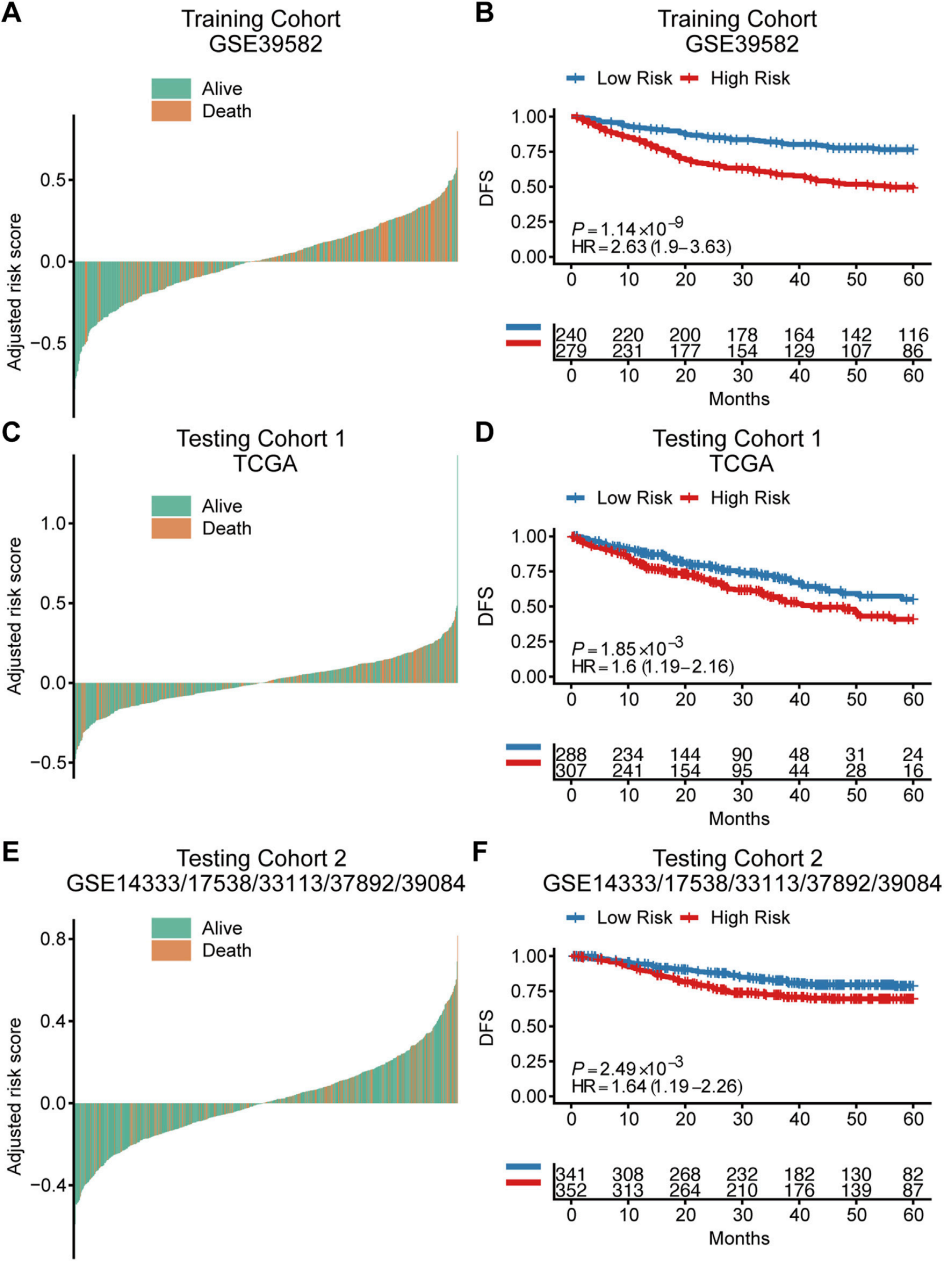
The prognostic value of the TRLs signature for colorectal cancer. Waterfall plots showed the distribution of survival status for patients of different TRLs signature risk groups in the training cohort **(A)**, testing cohort 1 **(C)** and testing cohort 2 **(E)**. Kaplan–Meier curves of DFS according to risk groups in the training cohort **(B)**, testing cohort 1 **(D)** and testing cohort 2 **(F)**. DFS: disease-free survival.

Based on the cut‐off value calculated by Youden index and the risk score of each patient, patients were divided into a high-risk group and a low-risk group in both training cohort and independent testing cohorts (Figures 2A,C,E). Kaplan-Meier curves with log-rank test (Figures 2B,D,F) and the univariate Cox regression analysis (Table 2) showed that the high‐risk patients had significant shorter disease-free survival (DFS) than the low‐risk patients in both training cohort (hazard ratio (HR) = 2.63, 95% confidence interval (CI) = 1.9–3.63, *p* < 0.001) and testing cohorts (testing cohort 1: HR = 1.6, 95% CI = 1.19–2.16, *p* = 0.002; testing cohort 2: HR = 1.64, 95% CI = 1.19–2.26, *p* = 0.002). Additionally, multivariate Cox regression analysis were also performed in the training and testing cohorts to examine whether the TRLs signature was an independent prognostic factor in CRC. Taking into consideration the risk group and other clinical or pathological parameters which were found significant in the previous univariate Cox regression, the results of multivariate Cox regression (Table 2) showed that risk group was an independent prognostic factor for DFS prediction in both training cohort (HR = 2.18, 95% CI = 1.53–3.12; *p* < 0.001) and testing cohorts (testing cohort 1: HR = 1.40, 95% CI = 1.04–1.91, *p* < 0.05; testing cohort 2: HR = 1.58, 95% CI = 1.14–2.18, *p* < 0.0001). It indicated that the TRLs signature was a promising predictor of prognosis for CRC patients, which had the potential to find clinical application.

**TABLE 2:** Univariate and multivariate Cox regression analyses.

	Univariate cox regression		Multivariate cox regression	
	Hazard ratio	*p*	Hazard ratio	*p*
Training cohort				
Risk group	2.63 (1.90–3.63)	1.10E-09	2.18 (1.53–3.12)	1.60E-05
Age	1.02 (1.00–1.03)	7.20E-03	1.01 (1.00–1.03)	3.20E-02
Sex	1.37 (1.01–1.85)	4.00E-02	1.44 (1.05–1.99)	2.50E-02
Stage	1.89 (1.52–2.34)	5.70E-09	1.77 (1.41–2.23)	1.10E-06
Location	0.95 (0.71–1.28)	7.50E-01	NA	NA
MSS/MSI	0.68 (0.42–1.10)	1.10E-01	NA	NA
CMS subtypes	1.19 (1.03–1.38)	2.00E-02	1.07 (0.92–1.25)	3.60E-01
Testing cohort 1				
Risk group	1.60 (1.19–2.16)	1.80E-03	1.40 (1.04–1.91)	2.90E-02
Age	1.01 (1.00–1.02)	2.20E-01	NA	NA
Sex	1.08 (0.81–1.45)	6.00E-01	NA	NA
Stage	2.15 (1.80–2.56)	9.00E-19	2.12 (1.78–2.53)	3.10E-17
Location	1.12 (0.83–1.49)	4.60E-01	NA	NA
MSS/MSI	1.13 (0.83–1.53)	4.50E-01	NA	NA
CMS subtypes	1.15 (0.98–1.36)	9.30E-02	NA	NA
Testing cohort 2				
Risk group	1.64 (1.19–2.26)	2.50E-03	1.58 (1.14–2.18)	5.70E-03
Age	0.99 (0.98–1.00)	1.90E-01	NA	NA
Sex	1.03 (0.75–1.40)	8.80E-01	NA	NA
Stage	2.31 (1.86–2.87)	3.00E-14	2.26 (1.82–2.81)	1.10E-13
Location	0.86 (0.57–1.28)	4.50E-01	NA	NA
MSS/MSI	0.92 (0.46–1.83)	8.10E-01	NA	NA
CMS subtypes	1.10 (0.94–1.29)	2.20E-01	NA	NA

Cox regression analyses were performed with DFS, data.

Location: location of tumor [right colon or left colon (rectum included)].

MSI, microsatellite instability; MSS, microsatellite stable; CMS, consensus molecular subtypes of colorectal cancer.

### The relationship between TRLs signature and immune pathways

**FIGURE 3:**
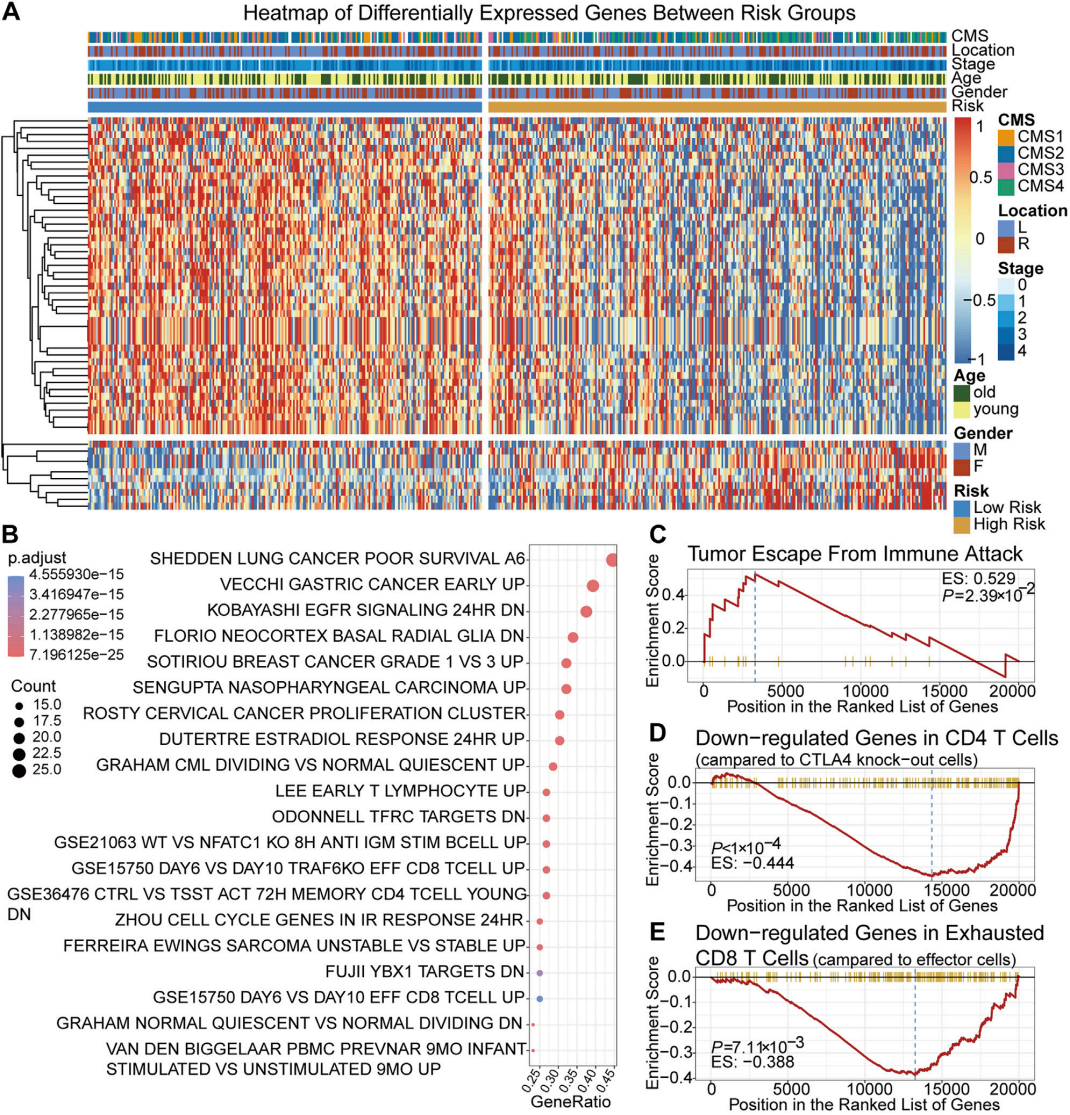
Identification and gene enrichment analysis of 56 DEGs between two risk groups. **(A)** A heatmap of 56 DEGs. **(B)** Bubble chart of the top 20 enriched MSigDB pathways of the DEGs. **(C–E)** Gene set enrichment plots of cancer immune escape related pathways and cancer immunotherapy related pathways. MSigDB pathways: C2, C5 and C7 pathways collection of the Molecular Signatures Database.

Between the low-risk group and high-risk group, 56 DEGs were identified. The expression of 56 DEGs, score group and corresponding clinical, molecular and pathological features of each patient were visualized with a heatmap and a volcano plot (Figure 3A; Supplementary Figure S2). Based on DEGs and the clusterProfiler package, the top 20 enriched pathways were shown in (Figure 3B). It showed that immune-related pathways involving CD8 positive T cells, CD4 positive T cells and T lymphocytes were among the top ones, suggesting that the TRLs signature risk score correlated closely with immune cells and immunity-related regulation. High-risk group were enriched in the pathway of tumor immune escape (Figure 3C), implying that immune escape might be one of the reasons contributing to the worse prognosis of the high-risk group. It was also found that low-risk group were enriched in genes sets that down-regulated in CTLA4 expressing CD4 positive cells and exhausted CD8 positive T cells, suggesting that patients of the low-risk group were not the potential target of immunotherapy (Figures 3D,E).

### The complex LncRNA-mRNA regulation network

**FIGURE 4:**
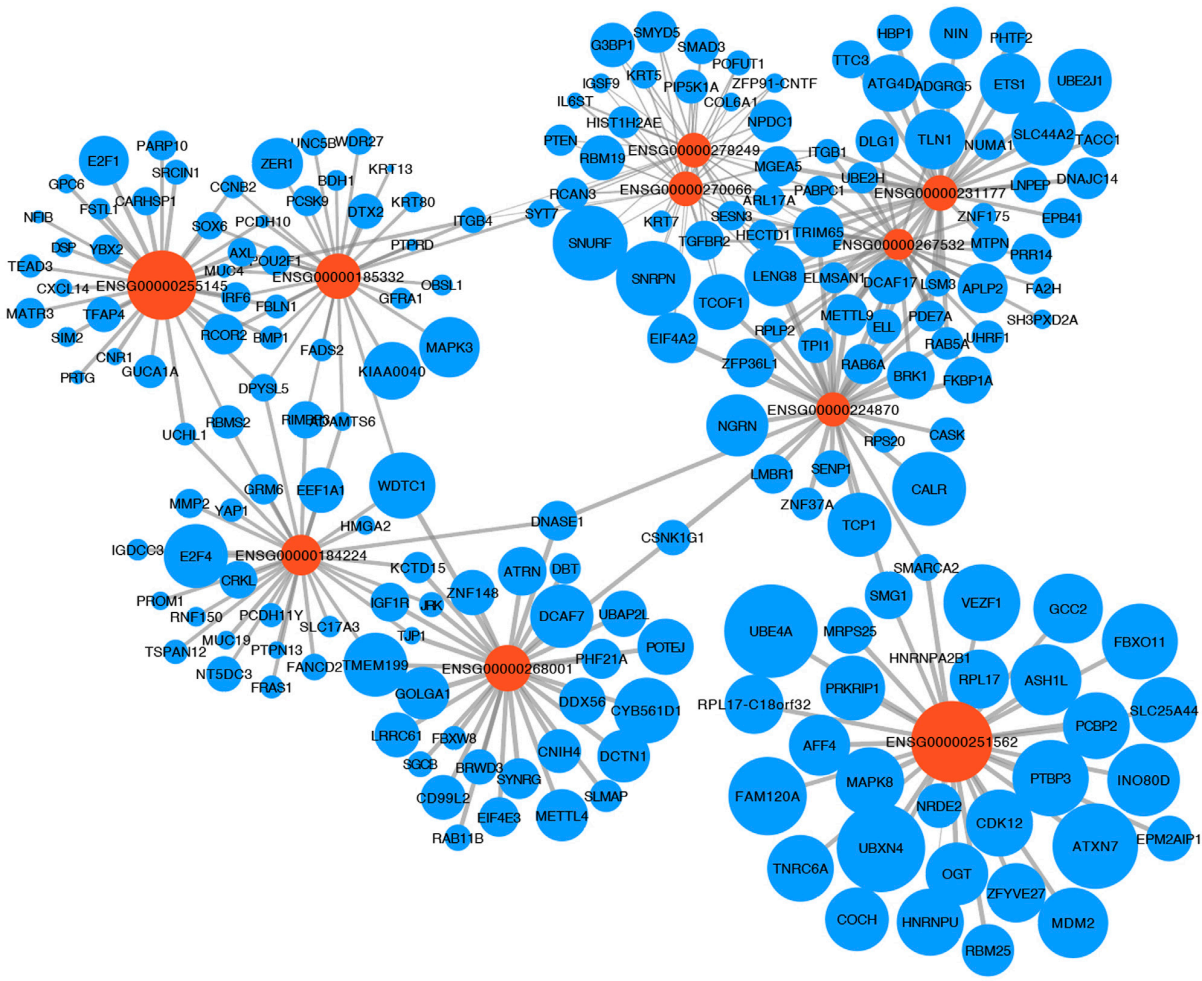
LncRNA-mRNA regulation network. The relationship between 10 TRLs of the signature (orange node) and their most correlated target mRNAs (blue nodes) was shown. The size of the nodes represented the average expression of lncRNAs and mRNAs in the immune cells, and the width of the lines represented the correlation between the expression of the lncRNAs and the expression of their targets.

Providing an insight into the complex regulatory mechanism of 10 TRLs of the signature, the most correlated lncRNA-target mRNA in the immune cells of the TIME were visualized with a network based on four manually-curated and experimentally-supported lncRNA-target mRNA interaction databases (Figure 4). Analyzing target mRNAs with GSEA, multiple pathways related to immunoregulatory mechanisms and immune cells were enriched, indicating that TRLs of the signature exerted a great impact on the TIME and tumor-related immune response (Table 3).

**TABLE 3:** Gene set enrichment analysis of TRLs targets.

LncRNA	Enriched Pathways/Pathways Related Cells
ENSG00000255145	IL-22 signaling; CD4 T cell; interferon; effector CD8 T cell
ENSG00000268001	T cell migration; lymphocyte migration
ENSG00000184224	Abnormality of the abdominal wall
ENSG00000185332	NKT cell activation; CD8 T cell
ENSG00000251562	IL-4 signaling; CD4 T cell; CD8 T cell; B cell; Treg cell; macrophage; monocyte; NK cell; dentric cell
ENSG00000267532	B cell; dentric cell; macrophage; monocyte; B cell
ENSG00000224870	IL-4 signaling; CD8 T cell; Treg cell; macrophage; B cell; CD4 T cell
ENSG00000231177	Memory CD8 T cell; naïve CD8 T cell; effector CD8 T cell; Treg cell; monocyte; B cell; CD4 T cell
ENSG00000270066	CD4 T cell; B cell; macrophage; interleukin 4/6/12/13/27/35/37 signaling; NKT cell; Treg cell; NK cell; dentric cell; CD8 T cell
ENSG00000278249	CD4 T cell; B cell; macrophage; interleukin 4/6/12/13/27/35/37 signaling; NKT cell; Treg cell; NK cell; dentric cell; CD8 T cell

### Tumor immune environment characterization

**FIGURE 5:**
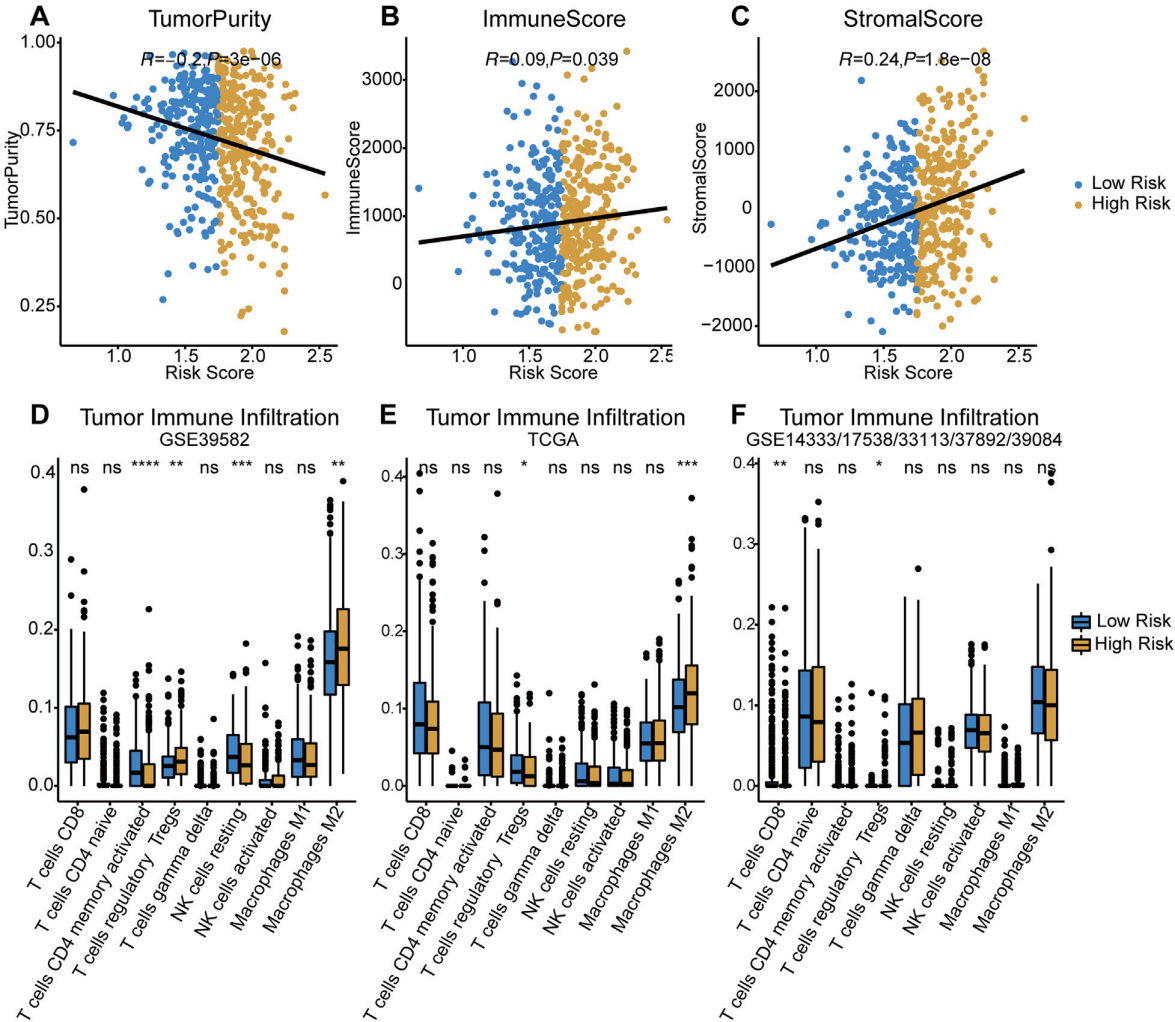
Evaluation of tumor immune infiltration in both risk groups. **(A–C)** Comparisons of tumor purity, immune score and stromal score between low-/high-risk groups. **(D–F)** Difference of tumor infiltrating immune cells in two risk groups among three cohorts. *p* < 0.0001 ****, *p* < 0.001 ***, *p* < 0.01 **, *p* < 0.05 *, not significant: ns.

Assessed with ESTIMATE algorithm, the infiltration level of stromal cells (StromalScore) and immune cells (ImmuneScore) were significantly higher in high-risk group (*p* < 0.05 and *p* < 0.001, respectively), while significant lower tumor purity was observed in the low-risk group (*p* < 0.001, Figures 5A–C). The results of CIBERSORT immune infiltration analysis showed that the fraction of M2 macrophages and Tregs was significantly higher in the TIME in both training and testing cohorts (Figures 5D–F). In summary, the tumor tissue of the high-risk group was associated with pro-tumor TIME and greater degree of pro-tumor immune cells infiltration.

### The TRLs signature prediction in colorectal cancer immunotherapy

**FIGURE 6:**
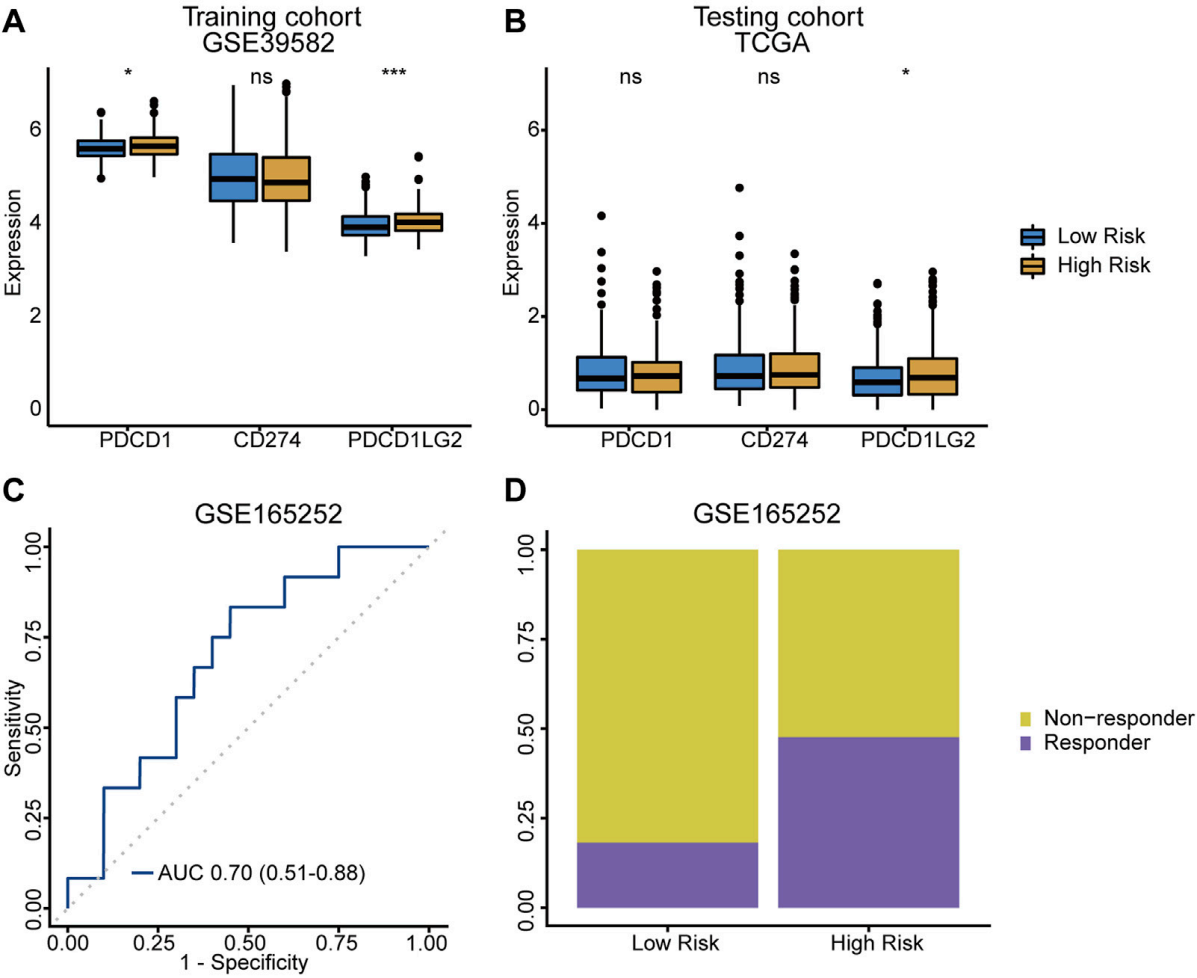
TRLs signature predicting immunotherapy response. **(A,B)** Difference of crucial immune checkpoint genes expression levels between low-/high-risk group. **(C)** The ROC curve for predicting anti‐PD-L1 immune checkpoint blockade therapy response of the TRLs signature. **(D)** Difference of anti‐PD-L1 immune checkpoint blockade therapy response rates between low-/high-risk groups. PDCD1: PD-1. CD274: PD-L1. PDCD1LG2: PD-L2. *p* < 0.001 ***, *p* < 0.01 **, *p* < 0.05 *, not significant: ns.

The expression of immunotherapy targets, such as PD‐1 (PDCD-1), PD‐L1 (CD274) and PD‐L2 (PDCD1LG2), were evaluated in both risk groups. Both PD‐1 and PD‐L2 were significantly upregulated in high-risk group (Figures 6A,B), suggesting the potential role of the TRLs signature in stratifying CRC patients for immune checkpoint inhibitor therapy. Meanwhile, immunotherapy dataset GSE165252, which was originally about atezolizumab (a PD-L1 inhibitor) treating esophageal adenocarcinoma, was used as an external dataset to verified the signature’s ability of making immunotherapy response predictions. As a result, an AUC value of 0.70 (95% CI = 0.51–0.88) was achieved (Figure 6C), and higher proportion of responders was also observed in the high-risk group (Figure 6D). The TRLs signature was capable of predicting immunotherapy response, suggesting that patients of the high-risk group would get more rewards from the anti-PD-1/PD-L1 therapy.

## Discussion

In recent years, lncRNAs have attracted extensive attention and there are a great number of studies about the relationship between lncRNAs of the TIME and characteristics of the tumor, including the prognosis, TME and anti-tumor immunity. A large number of lncRNAs are expressing different patterns in types of cancer including CRC (Bhan et al., 2017; Deng et al., 2017) and regarded as a vital player in tumorigenesis, anti-cancer immune response and immunotherapy (Yu et al., 2018). The TIME and its regulation are sculpted by tumor infiltrating immune cells, and lncRNAs of the tumor infiltrating immune cells plays an important role in this procedure (Denaro et al., 2019), indicating that the lncRNAs of CRC TIME has an unique value in prognostic and guiding patient stratification for immunotherapy. Here, a TRLs signature of CRC was established and verified in independent cohorts, providing distinct survival and immunotherapy response prediction for low and high-risk groups.

By forming a complex regulation network, TRLs of the signature modified the expression patterns of multiple target genes in the tumor infiltrating immune cells of TIME, especially genes related to immunoregulatory mechanisms and pathways. A previous study reported that lncRNA MALAT1 (ENSG00000251562 of the TRLs signature) promotes tumor angiogenesis in thyroid cancer by regulating functions of macrophage in the TIME (Huang et al., 2017). Due to the difference in the gene expression pattern between the high and low-risk groups controlled by multiple TIME lncRNA-based mechanisms, the infiltration, activation, function and fate of tumor infiltrating immune cells differ between the risk groups (Huarte and Rinn, 2010; Batista and Chang, 2013; Uthaya Kumar and Williams, 2020). Here, a significantly greater degree of pro-tumor immune infiltration was found in the high-risk group, especially M2 macrophage cells and Tregs, which impaired anti-tumor immunity, promoted tumor progression and contributed to tumor immune escape and poor prognosis (Bader et al., 2020; Zeng et al., 2020). As a result, profound changes in the niche the led to significant alterations in the gene expression profiles and behaviors of the tumor, bringing about different courses and outcomes of disease between low-risk group and high-risk group.

Having the power to rewrite the regulation network in the tumor-infiltrating immune cells and the TIME (Uthaya Kumar and Williams, 2020; Wells et al., 2020), lncRNAs also take part in controlling the immune surveillance, drug resistance and the efficacy of immunotherapy (Pi et al., 2021). Many studies have shown that immune-related lncRNAs were capable of predicting the response for immune checkpoint inhibitor therapy (Jiang et al., 2020; Sun et al., 2020; Ma et al., 2021; Xu et al., 2021; Zhou et al., 2021). Consistent with the reported findings, the CRC TRLs signature provided us with immunotherapy treatment indications, showing that patients of the high-risk group were associated with higher expression of cellular receptors targeted by immune checkpoint inhibitor therapy and favorable response towards immunotherapy, which is of great help to stratify CRC patients for immunotherapy (Bateman, 2021).

There are some limitations that should be acknowledged. First, as a retrospective study, the model was trained and validated on existing datasets, indicating that TRLs signature needs to be further validate on large prospective cohorts. Second, the major limitation of the study was the lack of experimental validation. Although the model performed well in survival and immunotherapy response prediction, the underlying biological functions of the signature’s TRLs and the complicated regulation mechanisms between TRLs and their target mRNAs the in the TME were not fully understood, which should be further studied with cellular and molecular experiments.

In summary, not only provides distinct survival prediction and insights into the TIME for the two risk groups, our TRLs signature also gives doctors with immunotherapy treatment indications, suggesting that the patients of low-risk group may have a chance to live longer and patients of high-risk group could benefit more from the immunotherapy.

## Data Availability

The datasets presented in this study can be found in online repositories. The names of the repository/repositories and accession number(s) can be found in the article/Supplementary Material.

## Glossary

lncRNA: long non-coding RNA
TIME: tumor immune microenvironment
CRC: colorectal cancer
TRL: tumor immune microenvironment related long non-coding RNA
LASSO: Least absolute shrinkage and selection operator
HR: hazard ratio;
CI: confidence interval
PD-L1: programmed cell death 1 ligand 1
PD-L2: programmed cell death 1 ligand 2
PD-1: programmed cell death 1
TME: tumor microenvironment
Treg: regulatory T cell
ceRNA: competing endogenous RNA
GEO: Gene Expression Omnibus
TCGA: The Cancer Genome Atlas
COAD: colon adenocarcinoma
READ: rectum adenocarcinoma
CCLE: Cancer Cell Line Encyclopedia
TSI: tissue specificity index
MMR: mismatch repair
MSS: microsatellite stability
MSI: microsatellite instability
CMS: consensus molecular subtypes
DEG: differentially expressed gene
GSEA: gene set enrichment analysis
MSigDB: Molecular Signatures Database
ROC: receiver operating characteristic
AUC: area under the curve
DFS: disease-free survival
CTLA4: cytotoxic T-lymphocyte associated protein 4.
