# Supplementary material for: Predicting prognosis and immunotherapy response among colorectal cancer patients based on a tumor immune microenvironment-related lncRNA signature
Source: Front Genet. 2022 Sep 7;13:993714. doi: 10.3389/fgene.2022.993714 (PMC9489948; doi:10.3389/fgene.2022.993714)
Supplement: Supplementary file 1 [file DataSheet1.docx]

Supplementary Material

## Supplementary Tables

| **Supplementary Table 1: Immune cells collected from GEO database** | | |
| --- | --- | --- |
| **Immune Cell Type** | **GSE** | **GSM** |
| T gamma delta | GSE13906 | GSM349848 |
| T gamma delta | GSE13906 | GSM349849 |
| Immature dendritic cell | GSE23371 | GSM573357 |
| Immature dendritic cell | GSE23371 | GSM573358 |
| Immature dendritic cell | GSE23371 | GSM573359 |
| Mast cell | GSE25320 | GSM622849 |
| Mast cell | GSE25320 | GSM622850 |
| Mast cell | GSE25320 | GSM622851 |
| Mast cell | GSE25320 | GSM622852 |
| T gamma delta non-activated | GSE27291 | GSM674835 |
| T gamma delta non-activated | GSE27291 | GSM674838 |
| T gamma delta non-activated | GSE27291 | GSM674841 |
| T gamma delta non-activated | GSE27291 | GSM674844 |
| NK cell | GSE27838 | GSM687236 |
| NK cell | GSE27838 | GSM687237 |
| NK cell | GSE27838 | GSM687238 |
| NK cell | GSE27838 | GSM687239 |
| NK cell | GSE27838 | GSM687240 |
| NK cell | GSE27838 | GSM687241 |
| NK cell | GSE27838 | GSM687242 |
| NK cell | GSE27838 | GSM687243 |
| CD4 T cell activated | GSE28726 | GSM711687 |
| CD4 T cell activated | GSE28726 | GSM710979 |
| CD4 T cell activated | GSE28726 | GSM711481 |
| CD4 T cell activated | GSE28726 | GSM711685 |
| CD4 T cell resting | GSE28726 | GSM710977 |
| CD4 T cell resting | GSE28726 | GSM711464 |
| CD4 T cell resting | GSE28726 | GSM711666 |
| CD4 T cell resting | GSE28726 | GSM711686 |
| NKT activated | GSE28726 | GSM711691 |
| NKT activated | GSE28726 | GSM711608 |
| NKT activated | GSE28726 | GSM711610 |
| NKT activated | GSE28726 | GSM711612 |
| NKT activated | GSE28726 | GSM711614 |
| NKT activated | GSE28726 | GSM711689 |
| NKT resting | GSE28726 | GSM711482 |
| NKT resting | GSE28726 | GSM711609 |
| NKT resting | GSE28726 | GSM711611 |
| NKT resting | GSE28726 | GSM711613 |
| NKT resting | GSE28726 | GSM711688 |
| NKT resting | GSE28726 | GSM711690 |
| Plasmacytoid dendritic cell | GSE37750 | GSM926937 |
| Plasmacytoid dendritic cell | GSE37750 | GSM926938 |
| Plasmacytoid dendritic cell | GSE37750 | GSM926939 |
| Plasmacytoid dendritic cell | GSE37750 | GSM926940 |
| Plasmacytoid dendritic cell | GSE37750 | GSM926941 |
| Plasmacytoid dendritic cell | GSE37750 | GSM926942 |
| Plasmacytoid dendritic cell | GSE37750 | GSM926943 |
| Plasmacytoid dendritic cell | GSE37750 | GSM926944 |
| Neutrophil | GSE39889 | GSM980737 |
| Neutrophil | GSE39889 | GSM980741 |
| Neutrophil | GSE39889 | GSM980745 |
| Neutrophil | GSE39889 | GSM980749 |
| Myeloid dendritic cell | GSE42058 | GSM1031685 |
| Myeloid dendritic cell | GSE42058 | GSM1031686 |
| Myeloid dendritic cell | GSE42058 | GSM1031687 |
| Myeloid dendritic cell | GSE42058 | GSM1031688 |
| B cell | GSE49910 | GSM1209554 |
| B cell | GSE49910 | GSM1209555 |
| B cell | GSE49910 | GSM1209556 |
| B cell | GSE49910 | GSM1209557 |
| Neutrophil | GSE49910 | GSM1209558 |
| Neutrophil | GSE49910 | GSM1209559 |
| Neutrophil | GSE49910 | GSM1209560 |
| CD8 T cell | GSE49910 | GSM1209564 |
| CD4 T cell | GSE49910 | GSM1209565 |
| CD8 T cell | GSE49910 | GSM1209566 |
| CD4 T cell | GSE49910 | GSM1209567 |
| CD8 T cell | GSE49910 | GSM1209568 |
| CD8 T cell | GSE49910 | GSM1209569 |
| CD8 T cell | GSE49910 | GSM1209570 |
| CD4 T cell | GSE49910 | GSM1209571 |
| CD8 T cell | GSE49910 | GSM1209572 |
| CD8 T cell | GSE49910 | GSM1209581 |
| CD8 T cell | GSE49910 | GSM1209582 |
| CD8 T cell | GSE49910 | GSM1209583 |
| CD8 T cell | GSE49910 | GSM1209584 |
| Monocyte | GSE49910 | GSM1209585 |
| Monocyte | GSE49910 | GSM1209586 |
| Monocyte | GSE49910 | GSM1209587 |
| Monocyte | GSE49910 | GSM1209588 |
| Monocyte | GSE49910 | GSM1209589 |
| Monocyte | GSE49910 | GSM1209590 |
| Dendritic cell | GSE59237 | GSM1431110 |
| Dendritic cell | GSE59237 | GSM1431111 |
| Dendritic cell | GSE59237 | GSM1431112 |
| Dendritic cell | GSE59237 | GSM1431113 |
| Dendritic cell | GSE59237 | GSM1431114 |
| Dendritic cell | GSE59237 | GSM1431115 |
| Dendritic cell | GSE59237 | GSM1431116 |
| Dendritic cell | GSE59237 | GSM1431117 |
| Dendritic cell | GSE59237 | GSM1431118 |
| Dendritic cell | GSE59237 | GSM1431119 |
| Immature dendritic cell | GSE6863 | GSM158184 |
| Immature dendritic cell | GSE6863 | GSM158186 |
| Immature dendritic cell | GSE6863 | GSM158187 |
| NK cell | GSE8059 | GSM198942 |

| **Supplementary Table 2. TLR signature risk score calculation (TLRs of the signature and the corresponding coefficients)** | |
| --- | --- |
| **TLR** | **coefficients** |
| ENSG00000255145 | 8.52E-02 |
| ENSG00000268001 | -8.11E-03 |
| ENSG00000184224 | 2.71E-01 |
| ENSG00000185332 | 1.27E-01 |
| ENSG00000251562 | 6.68E-02 |
| ENSG00000267532 | 1.13E-01 |
| ENSG00000224870 | -1.25E-02 |
| ENSG00000231177 | -3.32E-01 |
| ENSG00000270066 | -3.12E-02 |
| ENSG00000278249 | -1.37E-16 |
| TLR: TIME related lncRNA | |
| Risk score = ∑ ( TLR coefficient * TLR expression) | |

## Supplementary Figures


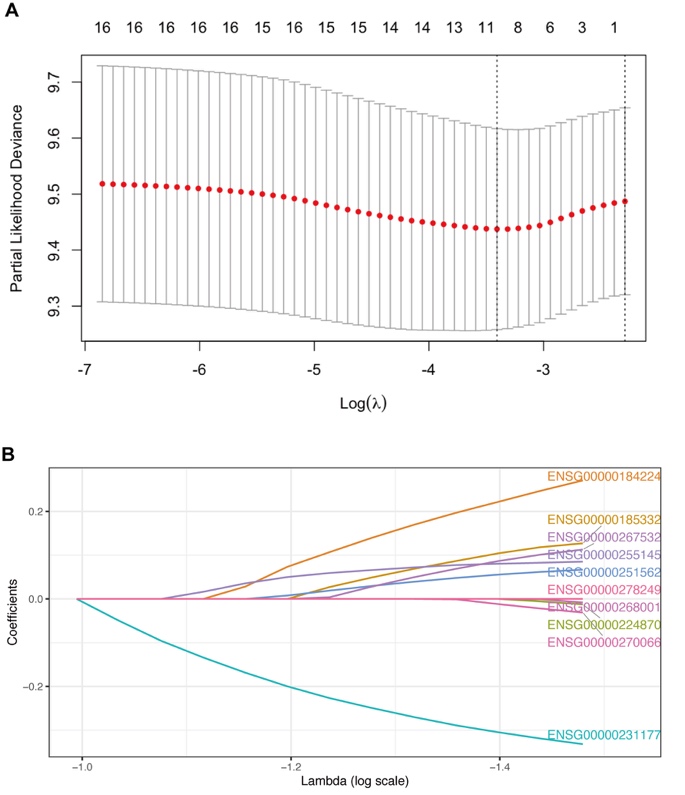


**Supplementary Figure 1.** The construction of TRLs signature in the training cohort. (A) Partial likelihood deviance of LASSO regression. (B) LASSO coefficient profile.


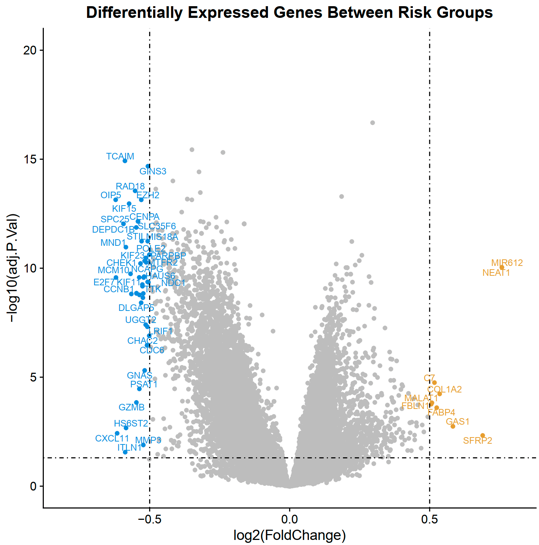


**Supplementary Figure 2.** The construction of TRLs signature in the training cohort. (a) Partial likelihood deviance of LASSO regression. (b) LASSO coefficient profile.
